# Regulating STING in health and disease

**DOI:** 10.1186/s12950-017-0159-2

**Published:** 2017-06-07

**Authors:** Yang Li, Heather L. Wilson, Endre Kiss-Toth

**Affiliations:** Department of Infection; Immunity and Cardiovascular Disease, University of Sheffield, Beech Hill Road, Sheffield, S10 2RX UK

**Keywords:** Stimulator of Interferon Genes (STING), Double-stranded DNA sensor, Cyclic dinucleotide, cGAS

## Abstract

The presence of cytosolic double-stranded DNA molecules can trigger multiple innate immune signalling pathways which converge on the activation of an ER-resident innate immune adaptor named “STimulator of INterferon Genes (STING)”. STING has been found to mediate type I interferon response downstream of cyclic dinucleotides and a number of DNA and RNA inducing signalling pathway. In addition to its physiological function, a rapidly increasing body of literature highlights the role for STING in human disease where variants of the STING proteins, as well as dysregulated STING signalling, have been implicated in a number of inflammatory diseases. This review will summarise the recent structural and functional findings of STING, and discuss how STING research has promoted the development of novel therapeutic approaches and experimental tools to improve treatment of tumour and autoimmune diseases.

## Background

Cellular stresses or infections lead to the release of DNA molecules into the cytoplasm which may threaten the stability of the host genome [1]. The intracellular appearance of naked DNA molecules triggers a double-stranded (ds)DNA sensing mechanism which consequently induces innate immune responses including the production and release of type I interferons (IFN-I). This response is central to the resolution of DNA-induced cellular stress [2–4] and prevents the emergence of autoimmunity [4, 5]. A recently described protein named STING (Stimulator of Interferon Genes, also known as TMEM173, ERIS, MITA and MPYS) is a critical regulator of these innate immune responses [6–9].

STING is an endoplasmic reticulum (ER)-resident transmembrane protein and was first recognised as part of the ER translocon system [6, 10]. Suppression of components of the translocon-associated protein (TRAP) complex such as TRAP*-*β and Sec61 β have been found to impair DNA-induced type I interferon (IFN-I) signals downstream of STING [11]. The TRAP complex has been shown to be involved in two of the ER’s major responses: protein N-glycosylation [12] and endoplasmic reticulum-associated degradation (ERAD) [13]. Although the functional relevance of STING in the ER translocon system has not yet been fully elucidated, it has been proposed that STING can interact with TRAP component Ssr2/TRAPβ to enable its migration from the ER to perinuclear membranes, a process key to IFN-β promoter activation [6, 14].

Recent reports have demonstrated that cytoplasmic DNA released by microbes and viruses can trigger dsDNA-sensing pathways which activate STING [6, 15–17]. STING then signals to the TANK binding kinase 1 (TBK1) / interferon regulatory factor-3 (IRF3) axis to upregulate type I interferon production [11, 18]. As an IKK (IκB kinase) –related kinase, TBK1 can also interact with IκB kinases to induce phosphorylation and thus degradation of IκB, thereby liberating NF-κB (nuclear factor kappa B) subunits allowing their nuclear translocation resulting in upregulation of type I interferon and other pro-inflammatory cytokines such as IL-6 (interleukin-6), CXCL10 (C-X-C motif chemokine 10), CCL5 (C-C motif chemokine ligand 5) and CCL2 [19]. Table 1 summarises the pathogens that have been shown to activate STING [6, 20–37]. Of note, STING knockout mice generated by Ishikawa and Barber were highly susceptible to infection by the single stranded RNA viruses vesicular stomatitis virus (VSV) and Sendai virus [6], suggesting STING activation pathways may overlap with RNA sensing mechanisms or reverse transcription of viral RNA [6, 24, 38].

**Table 1:** STING is activated by a range of pathogens

Name	Type of Pathogen	Mechanism of STING activation	References
Adenovirus	Non-enveloped linear dsDNA virus	dsDNA activates cGAS-STING pathway (cytoplasmic)	[20]
Kaposi’s sarcoma-associated herpesvirus	Enveloped dsDNA virus	dsDNA activates cGAS-STING pathway (cytoplasmic) and IFI16-STING pathway (nuclear)	[21]
Herpes simplex virus	Enveloped dsDNA virus	dsDNA activates cGAS-STING pathway (cytoplasmic) and IFI16-STING pathway (nuclear)	[22, 23]
Epstein-Barr virus	dsDNA virus	dsDNA activates IFI16-STING pathway (nuclear)	[24]
Human cytomegalovirus	dsDNA virus	dsDNA activates cGAS-STING pathway (cytoplasmic), DAI-STING (cytoplasmic), and IFI16-STING pathway (nuclear)	[25–27]
Sendai virus	Negative strand ssRNA virus	Possibly via RIG-I –dependent RNA detection which may in turn induce STING	[6]
Vesicular stomatitis virus	Negative strand ssRNA virus	Unknown	[6]
Human immunodeficiency virus	Negative strand ssRNA virus	dsDNA reverse transcribed from viral RNA induces cGAS-STING pathway	[28]
Influenza A virus	Negative strand ssRNA virus	Possibly via membrane fusion or unknown mechanism independent of DNA recognition	[29]
*Mycobacteria tuberculosis*	Bacteria producing c-di-GMP	c-di-GMP	[30, 31]
*Streptococcus pneumoniae*	Bacteria dsDNA	Bacterial dsDNA	[32]
*Streptococcus pyrogenes*	Bacteria dsDNA	Bacterial dsDNA	[33]
*Staphylococcus aureus*	Bacteria producing c-di-AMP	c-di-AMP	[34]
*Listeria monocytogenes*	Bacteria producing c-di-AMP	c-di-AMP	[35]
*Vibrio cholera*	Bacteria producing 3′-3′ cGAMP	3′-3′ cGAMP	[36, 37]

*Abbreviations: dsDNA* double-stranded DNA, *ssRNA* single-stranded RNA

The type I interferon signal adaptor protein STING is responsible for mediating double-stranded DNA sensing responses and the detection of bacterial cyclic dinucleotides c-di-AMP, c-di-GMP, and 3′-3′ cGAMP. Many DNA viruses, RNA viruses and bacteria have been implicated in the activation of STING

Whilst the STING-mediated dsDNA-sensing mechanism is critical for successful cellular protection against infections and disease progression, dysregulated STING activity leads to the excessive production of inflammatory mediators with potentially detrimental effects on surrounding cells and tissues. Recent studies revealed some important functions for STING in autoinflammatory diseases [39–41], cancer [41–44] and lipid regulations [45, 46], highlighting the importance of this protein in health and disease. Here we review the recent insights into STING function in human pathologies and discuss the potential of STING-targeted therapies which are of considerable scientific and clinical interest.

## Main text

### STING mediated signalling

#### Canonical STING activators

**Fig. 1:**
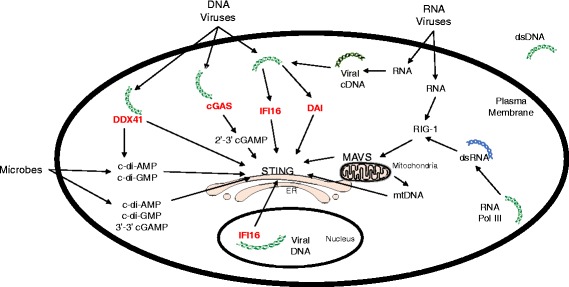
STING activation pathways. The endoplasmic reticulum (ER) adaptor STING is activated via recognition of bacteria-secreted 3′-5′ bond cyclic dinucleotides or DNA sensor cGAS-catalyzed 2′-5′ cGAMP. Cytoplasmic DNA, released from DNA viruses or reverse transcribed from the RNA viral genome, can induce direct interaction between STING and DNA sensors (*in red*) such as DDX41, IFI16, and DAI. Alternatively, RNA viruses also induce the RIG-I dependent MAVS activation which alters mitochondrial dynamics and indirectly induce STING activation. Mitochondrial stress can result in the release of mitochondrial DNA (mtDNA) which also induces DNA sensor activation (not shown) and STING-mediated signalling. RNA polymerase III (RNA Pol III) can convert dsDNA into dsRNA which activates RIG-I/ MAVS axis which has been shown to induce STING activation

Whilst STING acts as an adaptor protein in the dsDNA sensing pathway, it is not activated directly by DNA molecules. Instead, STING responds to DNA sensing proteins and molecules known as cyclic dinucleotides (CDNs) [35, 47–49] (Figure 1). CDNs are derived from infectious agents exogenously, or are produced by the mammalian dsDNA sensor cGAS (cyclic guanosine monophosphate – adenosine monophosphate synthase; cyclic GMP-AMP synthase). The canonical CDNs, or microbial secretory CDNs, are molecules made of 3′-5′ phosphodiester bonds joining two adenosines (A) – cyclic di-AMP [35, 50], two guanosines (G) – cyclic di-GMP [47] or one of each – cyclic GMP-AMP [37]. One of the STING-activating universally expressed DNA sensors, cGAS, is capable of catalysing a unique form of CDN endogenously upon DNA recognition [51]. This molecule is comprised of one 3′-5′ phosphodiester bond and a non-canonical 2′-5′ linkage between adenosine and guanosine, and is thus named 2′-3′ cGAMP to distinguish from the secretory cyclic dinucleotide cGAMP (3′-3′ cGAMP) which contains two 3′-5′ bonds [37]. Previous literature [52–54] has suggested that 2′-3′ cGAMP is ten- to a thousand-fold more potent than 3′-3′ cGAMP in activating STING. A number of studies reported that the change of phosphodiester linkage in 2′-3′ cGAMP results in a higher binding affinity to STING and thus leads to an augmented type I interferon response [55, 56]. It is also possible that hydrophilic secretory cyclic dinucleotides are excluded by the selectively permeable plasma membrane [57], and thus cannot be recognised by STING.

### Alternative STING activators

In addition to being activated by cyclic dinucleotides, STING also mediates antiviral responses downstream of DNA sensors including DNA-dependent activator of IFN-regulatory factor (DAI) [17], IFNγ-inducible protein 16 (IFI16) [15], DEAD-Box Helicase 41 (DDX41) [16], and components of the RNA-sensing pathways [23, 38] (Figure 1). The Z-form DNA sensor DAI was the first identified activator for STING, whose expression is highly cell-type and tissue-specific and therefore could not fully account for the widespread IFN-I induction during viral infection [17, 58]. Further research identified that the DNA sensor DDX41 could interact with bacterial cyclic dinucleotides, in addition to DNA molecules [16], prior to activating STING signalling [59].

Another interferon-inducible DNA sensor IFI61 is a pyrin-containing protein which also induces STING activation downstream of DNA detection. IFI16, as well as its mouse orthologue p204, is a universally expressed DNA sensor, which forms multimers prior to STING activation in response to HSV viral infections [15, 60]. Evidence shows that IFI16 is responsible for detecting foreign DNA both in the cytoplasm and in the nucleus and therefore it is capable of combating nuclear-replicating viruses such as Kaposi’s Sarcoma associated herpesvirus [61, 62] and human cytomegalovirus (HCMV) [27, 63], implying an ability to discriminate between “self” and “non-self” DNA molecules. A recent report by Diner and colleagues showed dynamic regulation of IFI16 oligomers at different cellular compartments in response to altered viral infections [64]. Throughout HCMV infection, IFI16 oligomers are densely gathered nuclear “punctate” structures, whereas in Herpes simplex virus-1 (HSV-1) infection these “puncta” become gradually dispersed across the whole nucleoplasm and are eventually degraded. In contrast to previous studies, Diner et al. also found that IFI16 knockout cells do not impede TBK1 activation upon immune stimulation, whereas both STING and cGAS knockout cells will strongly suppress TBK1 activity, suggesting that nuclear DNA detection mediated by IFI16 is independent of the STING/TBK-1/IRF3 axis. Other studies indicate that antiviral cytokine production occurs in the absence of IFI16 via an unknown mechanism [64].

Recent reports have revealed an interesting relationship between IFI16 and cGAS during DNA detection. It was shown that HSV infection can induce both DNA sensors in various cell types, and that cGAS is partially nuclear, thus is able to regulate the stability of nuclear IFI16 oligomers during detection of viral DNA [65]. This provides a molecular mechanism by which cGAS regulates nuclear DNA sensing. Further evidence also suggests a DNA dose-dependent interaction between IFI16 and cGAS in keratinocytes [66]. Although this interaction does not affect cGAS’s ability to generate cyclic dinucleotides, evidence indicates that IFI16 can facilitate the detection of these ligands by STING, and the loss of IFI16 can significantly impair downstream type I interferon and pro-inflammatory signalling [66]. Therefore, IFI16 and cGAS are not redundant during DNA infections, but instead cooperate and regulate each other’s activities.

### RNA-induced STING activation

Interestingly, several RNA viruses such as human immunodeficiency virus (HIV) [28, 67], influenza A virus [29], Sendai virus and vesicular stomatitis virus [6] have been found to activate STING signalling, via mechanisms both dependent and independent of DNA detection. Complementary DNA (cDNA) produced from reverse transcription of negative-stranded RNA in retroviruses such as HIV, murine leukemia virus (MLV) and Simian immunodeficiency virus (SIV) can induce a cGAS-dependent DNA sensing pathway and STING activation [28, 68] (Figure 1). However, HIV, in particular, is capable of inhibiting transcription of immediate anti-retroviral factors [69] and exploits the host STING blocker NOD-like receptor NLRX1 to aid the establishment of virus latency [67, 69]. It was also reported that cationic liposomes and nucleic acid-free herpesvirus-derived virus-like particles can directly induce STING/TBK1 relocation regardless of DNA sensing pathways, suggesting that the membrane fusion mechanism may be an alternative route for enveloped DNA and RNA viruses to activate STING. Enveloped RNA virus Influenza A virus has been shown to release haemagglutinin fusion peptide which induces STING but not cGAS activation [29], thus indicating another STING activation mechanism independent of cyclic dinucleotide recognition. It remains unclear whether the fusion particles alone are direct STING ligands or if activity requires facilitation by unidentified co-regulator(s).

The RNA-inducing adaptor MAVS (mitochondrial antiviral signalling protein; also known as VISA, Cardif, IPS-1) can also interact with and activate STING [6, 8] (Figure 1). The mitochondria-resident adaptor MAVS is the major molecular platform through which the RLR-dependent RNA sensing pathway elicits the type I interferon response. MAVS responds to the cytoplasmic RNA sensors RIG-I (the retinoic acid-inducible gene I) and MDA5 (melanoma differentiation-associated protein 5) [70], and in turn induces proinflammatory transcription factors NF-κB (nuclear factor kappa-light-chain-enhancer of activated B cells), IRF1, IRF3, IRF5 and IRF7 [70–73]. Castenier and colleagues [74] found that RIG-I induced MAVS activation can modulate mitochondrial dynamic changes promoting signalling to STING at MAMs, “mitochondria-associated membranes”, where mitochondria and ER are closely associated [75]. This adaptor interaction was found to be dependent on a mitochondrial fusion mechanism which induces mitochondrial elongation towards the ER, and hence MAM formation. Virus-induced mitochondria fragmentation disrupts membrane association and hence abolishes MAVS activation and secondary STING signalling [74]. Another report also suggests that RNA virus-induced release of stress-associated mitochondrial (mt)DNA activates the STING-dependent dsDNA sensing pathway [76]. This process, which is also likely to involve mitochondrial stress-induced apoptosis, may provide an effective means to remove damaged cells for infection control.

Ablasser [77] and Chiu [78] noted that the STING inducer and a B-form dsDNA sensor RNA polymerase III (Pol III) activate the RIG-I –dependent RNA sensing pathways (Figure 1). RNA Pol III reversely transcribes dsDNA into dsRNA molecules that are activators of RIG-I. However, it is unclear how STING is involved in this process.

### Post activation trafficking of STING

Activation of STING induces adaptor dimerization [7] and subsequent migration from ER membranes to punctate membranes of the Golgi by mechanisms similar to autophagy [11]. It remained difficult to identify the role of STING in autophagy regulation as little evidence was found to link STING with the recycling process during cell starvation. Yet many autophagy-related proteins such as autophagy-related gene (Atg) 9a and Vps34 are key to STING inter-organelle trafficking [79], and that the loss of early autophagy marker LC3 II can significantly impair the STING-dependent innate response to viral and bacterial infections [79–81]. This autophagy-like behaviour could also be related to the ER-originated pre-autophagosome formation, where STING is located [41].

### Signalling downstream of STING

**Fig. 2:**
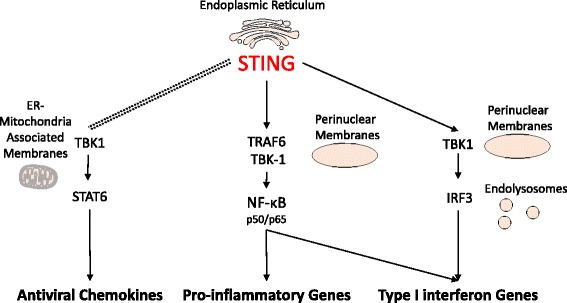
STING activated signalling pathways. STING activation leads to translocation from ER membranes to the perinuclear vesicles where it induces the signalling of two major pathways: the NF-κB -dependent proinflammatory response and the IRF3-dependent type I interferon response. The activation of mitochondrial antiviral adaptor MAVS also results in the activation of STING and recruitment of TBK1, which upregulates the transcription of antiviral chemokines via STAT6

The STING-dependent cellular responses are mainly dependent on two transcription factors, IRF3 and NF-κB (Figure 2). STING activation first induces adaptor dimerisation [82] and TRIM56-dependent ubiquitination to enable TBK1 docking [83]. Together, STING and TBK1 migrate to the perinuclear membranes of the Golgi via autophagy-like processes [6, 18, 79]. The association between STING-TBK1 leads to auto-phosphorylation of TBK1 at S172, a residue known to induce TBK1 activation [84, 85]. This further allows TBK1 to phosphorylate STING at S358 and S366 (S357 and S365, respectively, in mouse STING). Phosphorylated S366, together with L374, are important for the recruitment of IRF3 in close proximity to TBK1 at the C-terminus of STING, thereby enabling TBK1 to phosphorylate and activate IRF3 by exposing its nuclear localisation signal [18]. Activated IRF3 then translocates into the nucleus and promotes expression of type I interferons. Via a rapid feedback mechanism, IFN-I is released and binds to cell surface interferon receptors (IFNαRs), which then induces the expression of interferon-stimulated genes (ISGs) via Tyk2/JAK1 [86–88] and STAT1-STAT2 dimers [89, 90].

STING ligands have also been shown to activate the canonical NF-κB pathway (Figure 2), leading to the production of pro-inflammatory cytokines including IL-1α, TNF-α (tumour necrosis factor-α), IL-6, and numerous chemokines such as CXCL10 and CCL-2 [9, 19]. The mechanism of this was found to be dependent on STING-TBK1 activation, which in turn regulates the activation loop of IKKα/β releasing p65 to form active dimers with p50. Hence, the functional NF-κB complex can translocate into the nucleus and promote transcription of pro-inflammatory genes. Abe and Barber also suggested that TRAF6 may be involved upstream of TBK1, which likely facilitates NF-kB activation [19]. However, the IFI16-dependent STING pathway can only induce IRF3 but not NF-κB activation, which would serve to preserve the survival activities regardless of the antiviral response during infection [91].

## Regulatory motifs of STING activity

**Fig. 3:**
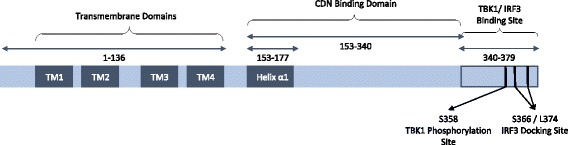
The domain structure of human STING protein. Human STING is a 379 amino-acid long ER-resident protein. The N-terminal contains 5 membrane-embedded domains (*dark blue*) including 4 transmembrane domains and Helix α1 responsible for ligand sensing and protein dimerisation. The C-terminal is mainly cytoplasmic (*pale blue*). It contains the cyclic dinucleotide (CDN) binding domain and interaction sites for TBK1 and IRF3 at the tail. Numbers above STING sequence indicate the amino acids comprising the functional domain

The sequence and topology of STING has been studied in parallel to its function. It is known that STING is a 379 amino acid long ER transmembrane protein encoded by the human *TMEM173* gene **(Accession NP_938023, XP_291127)** and homologous genes in other mammalian species. The STING structure is highly conserved between mammalian species, with the N-terminal forming a putative multi membrane-spanning region, a middle CDN-recognition domain, and a cytoplasmic tail (Figure 3).

It is understood that the N-terminal 130 amino acids of STING form four transmembrane helices [92, 93] that are mainly responsible for membrane anchorage and inter-organelle trafficking [94] (Figure 3). An additional helix, named helix α1, was previously considered as the fifth transmembrane domain [6, 8, 95, 96] but has more recently been proposed to form a distinct domain with different functions [97]. Helix α1 is formed between residues 153–177 and has been reported to play an essential role in protein folding and dimerisation. Compromising the integrity of helix α1 causes STING precipitation in the cytoplasm and abolishes the homo-dimeric structure due to the loss of abundant hydrophobic interactions between dimers [97]. Ouyang’s group reported that helix α1 also supports strong hydrogen force between STING dimers and cyclic di-GMP, suggesting its importance in multiple STING functions [98].

In addition to helix α1, the rest of the cytoplasmic tail also contributes to STING dimerisation [7], cyclic dinucleotide recognition [99], and TBK1 and IRF3 recruitment [18, 100] (Figure 3). In the absence of ligands, STING dimers show an open structure susceptible to cyclic dinucleotides [98]. Upon recognition of cyclic di-GMP, residues 139–344 are rearranged to expose a docking site for TBK1 [98], enabling TBK1 to phosphorylate STING at serine 358 [8]. This phosphorylation in turn enhances TBK1-STING attachment. Tanaka’s group found that a 39-residue fragment at the carboxyl end of the STING protein was sufficient to activate IRF3 signalling in response to DNA challenge [18], and the loss of this tail region, encoded by exon 7, creates a dominant negative STING isoform for TBK1-IRF3 signalling [101]. Further truncation of the STING C-terminal fragment revealed that S366 and L374 are the key residues for IRF3 recruitment and activation [18]. Therefore, TBK1 and IRF3 are recruited to STING in a 20 amino acid –spanning region to facilitate their interaction by close proximity.

Crystallisation of the STING protein has revealed that key residue substitutions or segment deletions significantly alter its structure, resulting in a dysfunctional protein [6, 7, 53, 97, 98, 102]. Mutations of single or multiple amino acids of STING have been found to influence its dimerisation capability [39, 103–105], ligand binding capacity [39, 104–106], or the ability to be post-translational modified by regulatory proteins [102, 107]. Some of these mutations occur naturally in humans to cause lethal autoinflammatory diseases [39, 105, 108], and some were generated experimentally in order to understand the structure-function relationship of STING [53, 96, 97, 102].

### STING variants

STING variations exist between mammalian species; the amino acid sequences of human and mouse STING are 68% identical and 81% similar [54]. Whilst this may not lead to dramatic differences in their three-dimensional structures, the structural differences at certain amino acid residues may be responsible for species-specific immune responses to some viral infections. For instance, Dengue virus (DENV) can inhibit STING signalling in human but not in mouse [107, 109]. DENV encodes for the protease NS2B3 that cleaves human STING at a highly-conserved putative cysteine motif (C88XXC91) to disable the adaptor. The equivalent cleavage target in mouse STING (mSTING) harbours a mutation that prevents NS2B3 cleavage, thus mouse STING can avoid DENV evasion. Mutant mouse embryonic fibroblasts transfected with hSTING re-constructed with a mouse NS2B3 cleavage site sequence blocks the type I interferon response against DENV. Specifically, a substitution of S135A in hSTING sequence results in inhibition of NS2B3 cleavage, suggesting that protecting STING at this residue may provide the basis for a novel anti-Dengue treatment [107].

In humans, single nucleotide polymorphisms (SNP) of STING were found to result in different levels of IFN-I signalling modulation (Table 2). Substitution at R284M can greatly stabilize the STING dimer, indicating its strong potential to cause chronic STING-dependent autoimmunity [103]. Substitution at R293Q can strongly reduce the c-di-GMP –induced IFN-I signals and completely abolishes the signals induced by other 3′-5′ bond cyclic dinucleotides [104]. Other single residue polymorphisms such as R232H and G230A may affect the ligand binding pocket of STING, thereby reducing its response to bacterial ligands [104]. In particular, a loss-of-function triple STING mutant, R71**H**-G230**A**-R293**Q** (named HAQ), abolishes almost 90% of the interferon response to all cyclic dinucleotides [110]. The HAQ mutant occurs in one-fifth of the population from a thousand genome screen [104]. Homologous HAQ mutant knock-in mice have demonstrated that a variety of immune cells, including lymphocytes and Ly6C^hi^ monocytes, express significantly less STING protein compared to the wildtype, and the mutant animals completely failed to respond to CDN challenge [106]. This suggested that the HAQ mutant may be a de facto null allele of STING, thereby reducing the availability of the STING protein to mediate a dsDNA sensing response.

**Table 2:** Mammalian STING variants and mutants

Mutation	Motif	Functional effect/ Disease association	Occurrence	References
S162A	ligand binding site	Reduce c-di-GMP binding Increase hSTING sensitivity to DMXAA	N/A	[53]
G230A	ligand binding site	Impair C-terminal binding to c-di-GMP	N/A	[104]
G230A-R293Q	ligand binding site	Double mutant Partially reduced IFN-β response to bacterial ligands	5.2% / 1000 human genome	[104]
R293Q	ligand binding site	Significantly reduced IFN-β response to bacterial ligands	1.5%/ 1000 human genome	[104]
R232H	ligand binding site	Partially reduced IFN-β response to c-di-GMP and complete loss of IFN response to other bacterial ligands	13.7% / 1000 human genome	[104]
R71H-G230A-R293Q (HAQ)	Recessive Null allele	Triple mutant Low intrinsic IFN-β/NF-κB promoter activity Homologous significant decrease STING expression and abolish IFN-I response to all STING ligands	20.4% / 1000 human genome	[104, 106, 110]
V155 M	Hydrophobic core, ligand binding site	SAVI, constitutive activation	Very rare	[39, 108]
N154S	Hydrophobic core, ligand binding site	SAVI, constitutive activation	Very rare	[39]
V147 L	Hydrophobic core, ligand binding site	SAVI, constitutive activation	Very rare	[39]
I200N	Interior STING promoter	Complete abolish STING activity, equivalent to I199N mSTING missense mutation, Goldenticket strain	N/A	[111]
G160E	Dimerisation domain	FCL, constitutive activation	Very rare	[105]
S366A (loss) or S366D (gain)	Ulk1/2 target phosphorylation site	Both loss-of-function (S366A) & gain-of-function (S366D) mutations block IRF3 binding	N/A	[102]

Single nucleotide polymorphisms of STING have been discovered in human and mouse which are implicated in dysregulation of type I interferon signalling and the proinflammatory innate immune response. STING mutations highlighted in green manifest as loss-of-function characteristics, mutations highlighted in red manifest as gain-of-function characteristics, and mutation in black lacks any gain-of-function or loss-of-function characteristic of STING. Gain-of-function mutations of V155 M, N154S and V147 L have been identified in human autoinflammatory disease called STING-associated vasculopathy with onset in infancy (SAVI), and substitution of G160E is the major cause of another human autoimmune disease known as familiar chilblain lupus (FCL). The most predominant loss-of-function STING mutant named HAQ is considered to compromise host innate response against infection, yet no clinical evidence is available for further discussion. Others STING mutations are experimentally created to study type I interferon signalling pathways, but they are potentially pathological

The function of several STING residues has been characterised using experimental point mutations (Table 2), some of which were also found to occur naturally in mammals. A C57BL/6 –derived, Goldenticket (Gt) mouse strain harbouring a single I199N mutation in STING leads to the complete abolishment of IFN-I activity to *Listeria monocytogenes* infection or stimulation of cyclic di-GMP and cyclic di-AMP [111]. The human equivalent mutation I200N was also considered to have the same effects, but no such spontaneous mutant has been discovered. Only a few gain-of-function hSTING mutants have been identified clinically [39, 105] (Table 2). Patients with these STING mutations showed early on-set of severe systemic inflammation in blood vessels and various organs, displaying chronic inflammatory symptoms that are highly similar to pathologies of SLE (systemic lupus erythematous) and AGS (Aicardi-Goutières Syndrome) [39, 105]. All of these STING mutants have shown considerable structural resemblance to the active conformation, presumably leading to constitutive adaptor dimerization and signalling to type I interferon production. Both Liu and König’s groups suggested that inhibition of the interferon signalling adaptor JAK could significantly dampen IFN-I over-expression as measured in biopsy samples from these patients, indicating that JAK inhibitors could be a promising avenue to therapeutically control disease progression.

As evidenced by the above studies, STING variants are likely to be associated with increased susceptibility to certain infections and autoimmune diseases, emphasising the value of genetic analysis of individual mutations to reveal novel targets for developing personalised therapy and immunisations.

## STING regulations

**Fig. 4:**
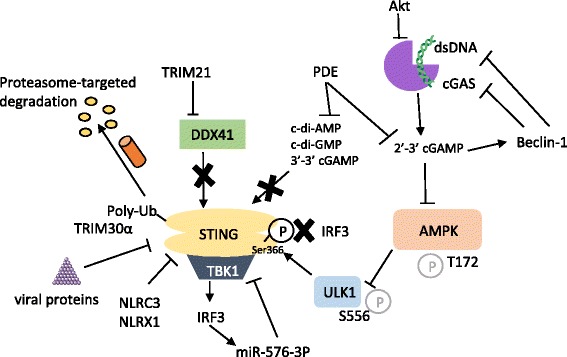
Negative regulation of STING-mediated response. STING-mediated signalling can be negatively regulated via multiple mechanisms, including E3 ubiquitin ligase TRIM30α- and TRIM21- mediated degradation of STING and its upstream DNA sensor DDX41, respectively. Certain phosphodiesterases (PDEs) also specifically hydrolyse bacterial cyclic dinucleotides to prevent them being sensed by STING. Akt kinase is also capable of inhibiting cGAS detection of cytoplasmic DNA. Activated cGAS produces 2′-3′ cGAMP to release AMPK-mediated inhibition of ULK1, which in turn blocks IRF3 recruitment downstream of STING activation. 2′-3′ cGAMP produced by cGAS can also activate Beclin-1 which can sequester cGAS as well as induce degradation of dsDNA. In a negative feedback loop, the product of the IRF3-dependent antiviral response, microRNA-576-3P (miR-576-3P), can prevent further STING activation. Some viruses can encode proteases or protein inhibitors to interfere in STING signalling, while others may enhance the activity of inflammasome complexes NLRC3 and NLRX1 to block STING/ TBK1 interaction

As a critical coordinator of the innate immunity, STING is tightly regulated by a variety of signalling molecules. Except that STING is post-translationally modified to enable dimerisation and activation, some regulators are essential for the prevention of constitutive type I interferon signalling which have been shown to cause autoimmunity both in animal models [6, 112] and in human [39, 108]. Negative regulation of STING signalling is necessary for the resolution of inflammatory responses post infection (Figure 4).

### Post-translational regulators

Post-translational modifications contribute to the spatio-temporal regulation of STING signalling. STING is commonly modified by ubiquitination and phosphorylation. Upon ligand binding, the E3 ubiquitin ligase TRIM56 is recruited to initiate K-63 linked ubiquitination on STING, a prerequisite for STING dimerisation and activation [83]. Another E3 ligase AMFR (Autocrine motility factor receptor), together with its interacting partner INSIG1 (insulin-induced gene 1), catalyses K-27 linked poly-ubiquitination, which is critical for TBK1 recruitment [113]. In contrast, TRIM30α-dependent K275 ubiquitination [112] and RNF5 (RING finger protein 5) -dependent K150 ubiquitination [114] degrade STING dimers and negatively regulate antiviral signalling (Figure 4). This process is likely to involve the antiviral adaptor protein MAVS on mitochondria at the MAMs where the closely associated ER and mitochondrial membranes bring STING and MAVS in close proximity [115, 116]. Since ubiquitination is critical to STING regulation, some viruses can secrete proteases to specifically disrupt this process to suppress innate immune recognition, as summarised below (Table 3).

**Table 3:** STING-mediated evasion of antiviral immunity

Pathogen	Mechanism of Action	References
Yellow fever virus Dengue virus	NS4B interrupts STING activation	[11]
Hepatitis C virus	NS3/4A, NS4B proteases interrupt STING activation	[137, 138]
Herpes simplex virus-1	Release ICP0 E3 ubiquitin ligase to degrade IFI16 Viral protein ICP27 binds to STING-TBK1 complex to prevent IRF3 signalling	[139, 140, 147]
Coronaviruses SARS and NL63	Disrupt K63-linked ubiquitin-mediated STING dimerisation	[140]
Human papillomavirus	E2 protein inhibits STING transcription E7 oncogene blocks cGAS/STING signalling	[141, 142]
Adenovirus	E1A oncogene blocks cGAS/STING signalling	[142]
Hepatitis B virus	Disrupts K63-linked ubiquitin-mediated STING dimerisation	[143]
Kaposi’s sarcoma-associated herpesvirus	ORF52 proteins bind to and inhibit cGAS Targets IFI16 degradation during lytic reactivation	[62]
Epstein-Barr virus, Murine gammaherpesvirus 68, Rhesus monkey rhadinovirus	ORF52 proteins bind to and inhibit cGAS	[144, 145]
Human immunodeficiency virus	Enhance STING suppressor NLRX1 Enhance TREX1 to degrade excessive cDNA Viral Capsids prevent innate sensing of cDNA	[67, 65, 68, 146, 148]
Human cytomegalovirus	Tegument protein pUL83 disrupts IFI16 oligomerization and activation	[63]

A number of DNA and RNA viruses have been found to encode and secrete STING-targeted proteases or inhibitors to prevent innate immune detection to help establish the of latent phase of infection. Viruses of the same family tend to adopt similar strategies/mechanisms to block STING activation. Some viruses also express multiple inhibitors to target both DNA sensors and STING, or release viral oncogenes in parallel to further compromise immunity, which consequently increases their chance of survival in the host

Some STING activities are also critically dependent on its phosphorylation. One such example is the phosphorylation of S366 on the C-terminal domain to provide docking sites for IRF3 prior to its phosphorylation by TBK1 [6, 100, 117]. ULK1-dependent phosphorylation on S366 post Golgi trafficking blocks IRF3 binding to STING and thus prevents chronic STING activation [102] (Figure 4). ULK1 acts upon release from its repressor AMPK (adenosine monophosphate activated protein kinase) which is induced by production of 2′-3′ cGAMP from cGAS. Interestingly, both loss-of-function (S366A) and gain-of-function (S366D) mutations can abrogate IRF3 signalling [18], suggesting that either the phosphorylation of S366 is temporally and spatially regulating IRF3 docking, or that alternative post-translational modifications are responsible for this functional regulation. In particular, S366-dependent inhibition of IRF3 does not impair the NF-κB pathway, indicating that these two pathways act independently of each other, which has likely evolved to prevent dysregulated antiviral responses from affecting survival activities [102].

STING can be negatively regulated by the NLR family inflammasome components NLRC3 [118] and NLRX1 [67] (Figure 4)*.* Both of these have been shown to sequester STING to prevent TBK1 recruitment; in particular the latter is strongly enhanced in HIV infection to suppress STING-dependent recognition of reverse-transcribed dsDNA [67]. Depletion of NLRX1 not only impedes nuclear transportation of viral DNA, but also restores the STING-mediated interferon response to stall progression of infection. This suggests that pharmacological suppression of intrinsic STING inhibitors could potentially support the re-establishment of STING-mediated innate immunity against RNA viruses, and thus may offer promising adjuvant therapy to combat retrovirus infection. However, Guo and colleagues also noted that such suppression must be finely controlled to avoid excessive inflammatory responses that may lead to autoimmunity [67].

### Post-transcriptional regulation

A primate specific microRNA (miR)-576-3p has been identified as a novel STING regulator that promotes virus replication [119] (Figure 4). Over expression of this microRNA promotes the spread of vesicular stomatitis virus, whereas its inhibition protects against virus growth. Further studies show that miR-576-3p is an IRF3-induced gene that can target multiple genes of interferon-stimulators, including STING, MAVS, TRAF3 and STAT6, thereby reducing their levels [119]. Since IRF3 is a downstream signalling molecule in the STING-TBK1 axis, upregulation of miR-576-3p serves as a negative feedback loop to prevent sustained inflammatory response during and post infections.

### Alternative mechanism of STING downregulation

A recent discovery suggests that certain phosphodiesterases (PDEs) may specifically degrade bacterial cyclic dinucleotides to halt excessive STING activation [120–123] (Figure 4). The pathogen *Mycobacterium tuberculosis* secretes phosphodiesterases MtbPDE (Rv3586), cnpB and CdnP to remove cytosolic CDN, thus avoiding STING mediated detection [124–126]. Although this may appear to undermine bacterial virulence and growth signals that are critical to infection [124], it can also significantly reduce early type I interferon induction. In particular, the enzyme CdnP can degrade both bacterial-derived and host-derived cyclic dinucleotides, critically promoting survival of *M. tuberculosis* at early stages of infection [125].

In addition to the above regulatory mechanisms, Akt kinase (or protein kinase B, PKB) has been shown to phosphorylate cGAS at residues S291 or S305 to stall signalling via STING [127], while the E3 ubiquitin ligase TRIM21 can specifically target the DNA sensor DDX41 at residues K9 and K115 for proteasome degradation and hence prevent recognition of DNA and STING-dependent type I interferon expression [128] (Figure 4). The autophagy protein Beclin-1 may also terminate STING-dependent immunity by sequestering cGAS and promoting autophagy-dependent digestion of dsDNA [129] (Figure 4). This is thought to prevent prolonged DNA recognition, which could lead to autoimmunity.

### STING in parasitic infection

Immune responses to malaria infection are highly strain specific; the lack of understanding linked to these strain specific responses makes the disease clinically difficult to manage [130–132]. Recent studies on host-parasite interaction have revealed distinct roles for STING-dependent type I interferon responses during crosstalk with other pro-inflammatory pathways. CD40 receptors expressed on antigen-presenting cells are understood to initiate the cellular and humoral response of the adaptive immunity, specifically enhancing the generation of immunoglobulins against pathogens [133, 134]. Mice infected with *Plasmodium yoelii nigeriensis,* infected red blood cells, TLR ligands or parasitic DNA/RNA upregulate both CD40 and type I interferon expression, whereas the loss of CD40 can reduce the level of STING further impairing the early type I interferon response in macrophages and dendritic cells [135]. Although CD40-induced STING upregulation leads to reduced CD40 levels and thus downstream NF-κB signalling, type I interferon immunity has been suggested to be highly inducible in certain strains of parasitic infection [135].

The establishment of malaria infection in the host is critically dependent on early innate immune mechanisms. Yu et al. suggested that depletion of plasmacytoid dendritic cells, rather than canonical dendritic cells and macrophages, can significantly impair type I interferon signalling at 24 h post *Plasmodium yoelii* infection [136]. The ubiquitous STING-cGAS pathway has been shown to enhance type I interferon production, and also potently induces SOCS1 to inhibit the MyD88 / IRF7-dependent type I interferon production which specifically acts on pDCs to protect against early malaria infection. The loss of STING and cGAS in mice augments the immediate type I interferon response from pDCs following malaria infection, and therefore protects the animal against early mortality [136]. Therefore, STING’s activity and its crosstalk with other proinflammatory pathways may be variable in complex diseases such as malaria, and thus the manipulation of STING signalling axis for therapeutic benefits may be difficult to achieve.

## Viral evasion of STING

It is evident that many viruses can evade STING signalling to establish a biological niche in mammalian cells, and that those within the same family tend to adopt similar evasive strategies [11, 62, 63, 65, 67, 68, 137–148] (Table 3). For instance, several members of the *Gammaherpesviridae* family were found to express ORF52 protein homologs to disrupt cGAS activities, preventing subsequent production of 2′-3′ cGAMP [48]. Species of the *Flaviviridae* and *Coronaviviridae* families tend to secrete proteases that directly cleave or block STING [140]; typical examples include the NS2B/3 and NS4B proteases released by *flaviviridae* Dengue virus and hepatitis C virus, respectively, which can both degrade hSTING. In particular, NS4B protease shows strong homology to the ER-embedded N-terminal domains of STING, leading to colocalisation and direct protein-protein interactions with STING [11]. It has also been suggested that tumour associated viruses such as human papillomavirus and adenovirus could potentially release oncogenic proteins to block cGAS/STING interactions with tumour suppressors, hence compromising innate immunity and supporting cancer progression [142].

The impairment or absence of interferon responses often seen in HIV infection has been proposed as one of the mechanisms by which this virus is capable of suppressing host immunity [148–151]. Recent research suggests that HIV-1 enhances the action of NLRX1 to dampen STING activity [67] and recruits host 3′ exonuclease TREX1 to degrade excessively produced, reverse-transcribed, viral DNA thereby avoiding detection by the cGAS/STING pathway [68, 152]. The capsid of HIV also regulates its association with host protein cyclin A, which controls the masking of viral cDNA from cGAS recognition in the cytoplasm and its exposure in the nucleus to facilitate genome integration [146]. Mutations in the HIV-1 capsid sequence enhance its binding to cyclin A prematurely in the cytoplasm enabling DC sensing of double-stranded DNA and a potent innate immune response against viral infection [146].

## STING related autoimmunity

Type I interferons are key cytokines induced by antimicrobial and antiviral immunity. This family of cytokines consists of the predominantly produced interferon-α and interferon-β, and the less abundantly expressed subtypes such as IFN-ε, −κ, −τ, and –ζ [153]. Type I interferons are ubiquitously expressed by a variety of cells including macrophages, lymphocytes, dendritic cells, fibroblasts and haematopoietic plasmacytoid dendric cells, with a widespread role in cellular biology [153, 154].

“Basal” expression of type I interferons is regulated via an autocrine mechanism [155], whereas the activation of interferon-inducing regulators such as STING can significantly boost their expression by activating the transcription factor IRF3. Type I interferons are released extracellularly for detection by self or nearby interferon receptors, IFNαRs, which are coupled to JAK1 (Janus kinase 1) and TyK2 (tyrosine kinase 2) [86, 87]. This activation further promotes the formation of STAT1-STAT2 heterodimers [23, 89, 90] and the subsequent recruitment of IRF9 to assemble the transcription complex ISGF3 to upregulates the expression of a series of interferon-stimulated genes (ISGs) [154]. A broad range of ISGs have been found to control chemotaxis, cell migration, apoptosis, cell proliferation, and to regulate immune detection and defense against infection; many of which have been thoroughly reviewed previously [156–160] (Table 4). Thus, dysregulation of type I interferon signalling can cause an excessive production of ISGs, in turn over-activating the immune system.

**Table 4:** Summary of interferon-stimulated genes (ISGs)

Apoptosis	Immune Modultion	Cell Attraction & Adhesion	Antiviral & Pathogen Detection
CASP4 CASP8 BAK1 Fas/ CD95 PLSCR1 XAF-1 DAP kinase RID	MxA MxB ICAM1 SELL CD47 ALCAM IRF1–5 & 7 LGALS3B IFN-γ IL-12 TNF-α SOCS USP18	VEGF FGF VRP PDGFRL ECGF1 EREG CTGF MHC I&II	OAS1 Protein Kinase R Viperin Tetherin IFI6 IFITM2 IFITM3 IRF1 IRF7 MHC I&II Ribonuclease L GTPase Mx1 ISG15 ISG20 ADAR1 APOBEC

Expression of ISGs are induced by type I interferon via the JAK-STAT signalling pathway. ISGs are involved in a wide spectrum of cellular activities including apoptosis, immune modulation, cell migration and adhesion, and antiviral responses. Some ISGs have multiple roles in immune regulation but are not repeatedly indicated in the table

The persistent or excessive presence of cytoplasmic DNA is one of the major causes for chronic inflammation and autoimmune diseases. Chronic production of type I interferons, termed “type I interferonopathy”, is a key indication of immune dysregulation predominantly associated with DNA-induced autoimmunity. The overactive IFN-I response alerts cytotoxic immune cells systemically via ISG production. This in turn promotes sustained release of proinflammatory cytokines including IL-1α/β, IL-12, and TNF-α, causing excessive inflammation and tissue damage [161–163]. Autoimmunity also induces aberrant cell death, which releases cellular components to T and B lymphocytes and leads to the production of self-reacting antibodies that congest in capillaries [164]. Unresolved B cell activation predisposes individuals to the development of systemic lupus erythematosus (SLE) that is clinically challenging to treat [152, 165, 166].

**Fig. 5:**
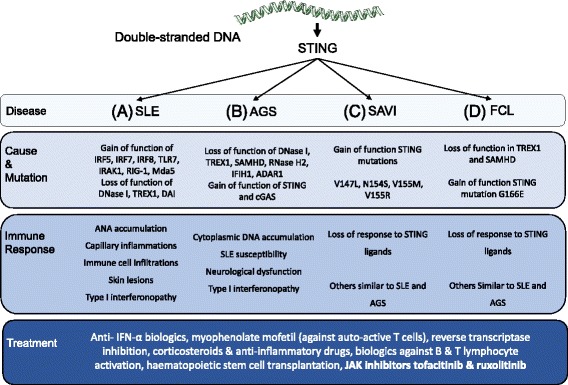
Cells and cytokines involved in STING-associated autoimmune diseases. Unresolved accumulation of cytoplasmic DNA can potentially trigger chronic inflammatory responses which result in autoimmune diseases, including **a** systemic lupus erythematosus (SLE) and **b** Aicardi-Goutières Syndrome (AGS). Both diseases are strongly associated with persistently enhanced type I interferon upregulation named type I interferonopathy and subsequent B and T lymphocyte activation that potentiates systemic tissue and organ damage. Though STING dysregulation has been suggested to play an essential role in the development of these diseases, current treatment of SLE and AGS still relies heavily on anti-inflammatory therapies and DNA resolving methods to ameliorate symptoms. Gain of function mutations of STING can cause two autoimmune diseases named **c** STING-associated vasculopathy with on-set in infancy (SAVI) and **d** familial chilblain lupus (FCL). Both diseases show similar manifestations to SLE and AGS and are much less responsive to STING ligands than other immune stimuli. Treatments for SAVI and FCL are limited but JAK inhibitors have been shown to ameliorate symptoms in patients with these two diseases

Systemic lupus erythematosus is a systemic chronic autoimmune disease. [152, 165, 166] (Figure 5). SLE is often diagnosed by the accumulation of serological antinuclear antibodies (ANA) against nucleic acids released from dead cells, which cause multiple tissue and organ damage [167]. Chronic activation of DNA and RNA sensing pathways triggered by infection and cell death can contribute to the type I interferonopathy that predisposes individuals to SLE [166]. Mutations in nucleic acid sensors (such as endosomal Toll-like receptors [168], RIG-I [169], and DAI [170]), interferon regulatory factors [171] and DNases [172, 173] can also increase SLE susceptibility. Recently, Ding and colleagues proposed that the cGAS-STING axis could be another pathway potentially exacerbating SLE, via the upregulation of type I interferon production downstream of cytoplasmic DNA sensing cascades [174]. However, STING deficiency in macrophages in fact renders hyper-responsiveness to endosomal TLR ligands, and STING knockout mice have shown accelerated lymphocyte accumulation and expansion of an IFN-α -responding cell population [175]. This therefore suggests inhibitory roles for STING in SLE development. Sharma et al. also showed that STING suppression can restrict the expression of regulatory T cell activation factor IDO-1 and TLR negative regulators such as A20, SOCS1 and SOCS3, contributing to uncontrolled systemic inflammation [175]. Since this effect is not seen in cells lacking IRF3, the transcription factor mediating most of DNA sensing responses downstream of the STING-TBK1 axis, it is possible that STING is immunosuppressive in inflammatory pathways independent of cytoplasmic DNA recognition. Therefore, autoimmune therapies targeting the STING pathway should be considered with caution and an awareness of the resultant STING down-regulation, which may have opposing effects in certain diseases.

AGS (Aicardi-Goutières Syndrome) is another genetically-based autoimmune disease, characterised by DNA-triggered type I interferonopathy [176] (Figure 5). Patients with AGS often carry mutations in DNA restriction factors including the 3′ exonuclease TREX1 [177, 178], dNTP restriction factor SAMHD (SAM domain and HD domain) [179, 180], RNase H2 (ribonuclease H2) [181, 182], dsRNA sensor IFIH1 (IFN-induced helicase C domain containing protein 1) [183, 184], and the dsRNA-specific adenosine deaminase ADAR1 [185]. These regulators maintain a balance between the production and degradation of nucleic acids, providing intrinsic protection against immune activation due to “self-recognition”. Mutations of DNA or RNA restricting factors cause nucleic acids to accumulate in the cytoplasm leading to SLE and AGS. Recent studies show that the type I interferonopathy associated with SLE and AGS is potentially cGAS-STING dependent, and that the aberrant IFN-I response can be suppressed by the loss of DNA sensor or STING in cells or animals expressing mutated *Trex1* or *Samhd* [180, 186].

Gain-of-function mutations in *STING* have been identified in infants who suffer from severe and chronic vasculopathy and pulmonary inflammation, a condition known as STING-associated vasculopathy with onset in infancy (SAVI) [39] (Figure 5). Mutations of STING V147 L, N154S, V155 M, and V155R were found to direct it to an active conformation enhancing dimerisation and inducing TBK1-IRF3 signalling (Table 2). This results in an excessive IFN-I response in fibroblasts, keratinocytes and immune cells to attract and amass proinflammatory cells and regulators in capillaries and tissues, ultimately causing lesions in these regions. Sustained IFN-I signals activate interferon receptors and promote expression of interferon-stimulated genes via JAK1-Tyk2 signalling and STAT1-STAT2 dimers. In vitro experiments and pioneering clinical studies suggest that JAK adaptor inhibition effectively dampens STING-mediated IFN-I over-activity. For instance, the elevated IFN-I levels in biopsy samples from SAVI patients can be restored close to that of the normal controls with treatment using the JAK inhibitor, tofacitinib [39]. Further investigation is required to examine the potential adverse effects of interferon suppression, which is likely to increase host susceptibility to infection.

Gain-of-function mutations of STING (Table 2) have also been linked to the autoimmune disease familial chilblain lupus (FCL) [105, 108], a rare hereditary form of SLE commonly associated with cytoplasmic DNA accumulation in monogenic mutations of exonucleases TREX1 [187, 188] or SAMHD [189] (Figure 5). A recent discovery reports FLC in five members of a four-generation family sharing the same *TMEM173* (STING) variant that encodes a single polymorphism of G166E. Structural analysis of mutated STING dimers reveals strong hydrogen attractions between E166 on one monomer and two threonine residues on the associating monomer, hence leading to enhanced adaptor dimerization and constitutive IFN-I -activated signalling [105]. Although limited treatment data is currently available for FCL, the authors showed that continuous administration of the JAK inhibitor tofacitinib can markedly ameliorate type I interferonopathy and associated symptoms in two patients. A similar therapeutic strategy was previously proposed by Liu and colleagues for treatment of STING-associated vasculopathy with onset in infancy (SAVI) [39]. Therefore, this therapeutic approach, based on mechanistic data, could be adapted to treat type I interferonopathy found in various diseases.

### STING regulates lipid metabolism

A new insight into STING research was recently provided by York and colleagues who suggested that this protein is a crucial element of cholesterol metabolism [45]. Previous studies indicate that high cholesterol levels in the plasma membrane correlate with viral loads and host susceptibility to infections [190, 191]. Virus and microbial infections have been shown to modulate lipid metabolism in the plasma membrane to facilitate infectivity. For instance, influenza virus encodes fusion protein haemagglutinin that is specialised in manipulating membrane lipid to permit penetration into the cytoplasm, a central step to viral infectivity and survival [192]. In addition, membrane lipids can also form signalling microdomain named lipid rafts which are frequently hijacked by HIV for attachment, signalling and budding to further promote infection [193, 194]. The antiviral type I interferon response reduces cholesterol availability in membranes to prevent viral infection; however the underlying mechanism remains largely unknown [195–197]. York’s group recently identified that STING/TBK1 signalling is critical to the production of type I interferons to reprogram lipid biosynthesis in pathogenic infection [45]. They demonstrated that the shift from lipid biosynthesis to lipid uptake not only affects the plasma membrane but also ER membranes, a cue to activate STING, bypassing the dsDNA sensing pathway. However, since STING is not the only adaptor for innate immunity against pathogenic DNA in the cytoplasm, it is conceivable that additional DNA sensors may further enhance STING actions to modulate cholesterol metabolism and promote antiviral processes. In light of the membrane fusion theory that potentially mediates STING activation [198], virus-host lipid regulation at the plasma membrane offers a promising and novel future research direction.

## Therapeutic targeting of the STING pathway

Studies on STING regulatory pathways provide novel insights into antiviral and anti-inflammatory therapies. Activating STING-dependent pathways has been developed therapeutically for antiviral and, more recently, anti-tumour benefit. The predominant approach taken has been to introduce STING ligand cyclic dinucleotides to promote the IFN-I response, to combat infection or to prevent tumour progression. In contrast, type I interferonopathy associated with STING over-activity represents another set of pathologies underlying autoimmunity. Counteracting these disease processes requires potent suppression of STING signalling; attenuation of the interferon receptor adaptor JAK is used as a current target, whilst inhibiting immune cell activation may also help ameliorate symptoms.

### Anti-tumour immunotherapies

Certain types of cancer cells express molecular structures specifically recognised by CD8α + dendritic cells (DCs), which subsequently interact with cytotoxic T cells to induce cancer cell death. This event, known as T cell priming, is a prerequisite for anti-tumour adaptive immunity relying on activation of CD8α + dendritic cells to promote IFN-I signaling in immature T cells [199]. However, cancer cells also boost anti-inflammatory immune cells and regulatory T cells (Treg) to restrict CD8α + DC activity thereby attenuating the activation of tumour-suppressive T cells [200, 201]. Therefore, a potent and long-acting adjuvant that can promote CD8α + DC activities is highly desirable to enhance T cell priming and subsequent anti-tumour immunity.

It has been reported that the IFN-I response critical to T cell priming during tumourigenesis is dependent on the cGAS / STING pathway [42, 43]. Loss of STING in dendritic cells abolishes antigen cross-presentation from CD8α + DC to T cells, whereas neither MyD88 nor TRIF knockouts can significantly affect DC IFN-I signalling, suggesting that STING may be the only adaptor central to this process [43]. In both immunogenic and irradiation-induced tumour models, tumour-derived DNA was engulfed and recognised by the universal DNA sensor cGAS prior to STING activation [42, 43]. However, direct stimulation with STING ligands also enhanced DC production of type I interferons, suggesting that STING-inducing therapies may offer potential as anti-tumour adjuvants.

Preclinical studies published recently suggest that DMXAA-derived cyclic dinucleotides have been successfully applied in established mouse models of malignant tumours achieving sustained tumour regression [202]. In malignant tumour B cells, STING also induces an ER stress response through the IRE-1 / XBP-1 (X-Box binding protein 1) pathway [203]. In the presence of 3′-3′ cGAMP STING dimers are phosphorylated and aggregate, rather than undergoing degradation. This consequently enables prolonged STING signalling to induce apoptosis of tumour cells. Similarly, Tang et al. showed administration of 3′-3′ cGAMP induces rejection of chronic lymphotic leukemia in mouse models [203]. Another promising vaccine candidate is the recently developed STINGVAX which combines granulocyte-macrophage colony-stimulating factor (GM-CSF) and formulated 2′-5′ – 3′-5′ linked cyclic dinucleotide [204]. In various established in vivo tumour models, STINGVAX has shown notable positive effects on dendritic cell activation and in promoting tumour-infiltrating T cells. Interestingly, activated cytotoxic T cells also upregulate the expression of PD-L1 (programmed death ligand 1), which enhances the therapeutic action of pro-apoptotic ligand PD-1 to promote tumour cell death. Although these STING-based vaccines have recently been developed and have been tested in mouse models, the synergistic effect of the combined STING agonist and immune promoting therapy represents a novel strategy to combat tumours by reinforcing both adaptive immunity and anti-tumour targets.

### JAK inhibitors ameliorate STING mutated autoimmunity

Inhibition of STING signalling can be achieved by targeting interferon receptors. As previously mentioned, gain-of-function mutations in *TMEM173* (the gene encoding STING) underlie type I interferonopathies that manifest in the autoimmune diseases, SAVI [39] and FCL [105]. Constitutive STING signalling was detected in both of these diseases resulting in dysregulated IFN-I signalling via interferon receptors and the adaptors JAK and Tyk. This leads to the accumulation of activated STAT1/2 dimers in the nucleus, promoting transcription of interferon-stimulated genes. Both Liu and König’s groups demonstrated that treatment with the JAK1/3 inhibitor tofacitinib in patient biopsy samples suppresses STAT activation and restores the STING response to immune stimuli similar to healthy control cells [39, 105] (Figure 5).

Following the work of Liu and colleagues, Fremond’s group conducted an 18-month clinical investigation on three patients expressing STING-mutations to assess the efficacy of the JAK1/2 inhibitor ruxolitinib [205] (Figure 5), which was previously found to partially inhibit STAT activation in in vitro studies of STING mutants [39]. Marked amelioration of systemic inflammation and reduction of interferon-stimulated gene expression was consistently observed in all three patients and in the subsequently recruited additional four patients with *STING* gain-of-function mutations. However, suspension of JAK inhibition in one patient resulted in a dramatic inflammatory relapse, though rescued by re-introducing ruxolitinib, demonstrating that this approach may be unsustainable and requires continuous monitoring [105, 205, 206]. Nonetheless, it is arguable that the partial inhibition of JAK-STAT pathway has the advantage of preserving STING-dependent immune protection against infection in these patients, since no excessive infection incidents were observed during these clinical trials [205]. Thus, it is timely to determine whether such JAK inhibitors can be modified and adapted for future treatment of type I interferonopathy.

### Anti-inflammatory biologics

Anti-inflammatory biologics represent a further opportunity to suppress interferon responses in order to control type I interferonopathy in autoimmune diseases, including but not limited to SLE, a major manifestation linked to STING over-activity. For instance, the anti-IFN-α drug sifalimumab is effective in controlling cutaneous and joint pain in SLE patients [207] (Figure 5).

Another approach to control SLE in STING gain-of-function mutations is to deplete or inhibit B-cell responses to prevent the over-production of auto-antibodies. The effective anti-SLE biologics, belimumab, targets the BLyS protein of B lymphocytes, preventing B cell activation and expansion critical to the production of autoantibodies and downstream activation of T lymphocytes [208, 209] (Figure 5). A series of stage II and stage III clinical trials have shown effective B-cell inhibition and significant improvement of clinical symptoms in combination with traditional care therapy of SLE in the treatment group compared to placebo control, whilst drug tolerance and immunosuppression-induced infection susceptibility were not markedly increased [209–211]. This suggests an effective drug efficacy and safety of belimumab over another B-cell depleting drug rituximab, which failed to ameliorate SLE symptoms in phase II and III trails [212]. Unlike JAK inhibitors and anti-IFN biologics, B-cell targeted therapies are much less effective in controling type I interferonopathy that consequently cause complex inflammatory responses in *STING* mutant patients. However, they are still commonly used to treat the SLE-related consequences of STING over-activation to stall symptom deterioration, while they have also been considered suitable adjuvant candidates for STING-targeted therapeutics.

### Delivery of STING ligands

Accumulating evidence link STING-mediated IFN-I signaling to anti-tumour activity, and thus STING ligands have been proposed to offer promising immune-enhancing therapies to defend against DNA infections and tumourigenesis [41, 213]. However, targeting intracellular proteins such as STING remains challenging as the plasma membrane is a highly hydrophobic and size-selective barrier that resists passive entry of chemicals [57]. In contrast to the de novo STING ligand 2′-3′ cGAMP [48], other cyclic dinucleotides are produced exogenously by pathogens and introduced into the cytoplasm by active transport or particle fusion [47, 50, 214]. Since the hydrophilic phosphate groups in dinucleotide compounds are strongly repelled by membrane lipid bilayers, designing an effective delivery system for cyclic dinucleotides would provide a significant step forward in the development of STING-specific therapeutics.

**Fig. 6:**
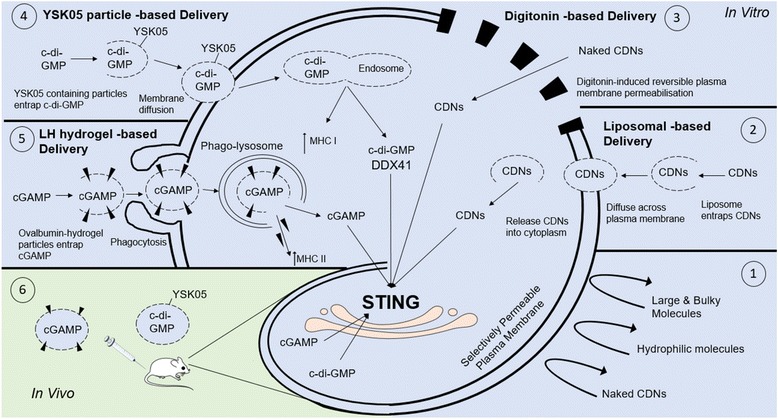
*In vitro* and in vivo delivery of STING agonists. The plasma membrane is a selectively permeable barrier that prevents cytoplasmic entry of large or hydrophilic molecules, including naked cyclic dinucleotides (CDNs) (1). *In vitro* (*blue background*) delivery of dinucleotide compounds could be achieved by the liposomal delivery system (2), or via reversible permeabilisation of plasma membrane to allow diffusion of naked CDNs into the cytoplasm (3). Recently designed YSK05-containing liposomes (4) could carry c-di-GMP across plasma membranes to induce DDX41-mediated STING activation as well as enhance the expression of MHC class I molecules and T cell co-stimulatory receptors (not demonstrated), and thus it is considered to be a potential adjuvant for cancer immunotherapy. In addition, the polyethyleneimine/ hyaluronic acid (LH) hydrogel-based vesicles use phagocytosis to deliver both STING ligands and antibody-stimulating agents such as ovalbumin (*dark triangles*) to cells (5), and enhance both STING-dependent innate immunity and MHC class II-activated adaptive immunity to suppress cancer growth. Both YSK05 particles and LH hydrogel-based particles have been tested in vivo (*green background*) to stall tumour progression in mice (6)

Under in vitro studies, transfection of microbial cyclic dinucleotides is aided by digitonin [35, 39, 50, 104] or liposomal-based systems such as Lipofectamine® [47, 102] (Figure **6**). The former method, first described by Woodward’s group [35], aims to achieve reversible permeabilisation of cellular membranes to increase uptake of chemicals [215–217], but is limited to in vitro studies due to high toxicity in vivo. In contrast, the liposomal-based delivery system is based upon the principle of encapsulating drugs in artificial double-layered liposomes for cytoplasmic delivery via liposomal fusion. The system is highly adapted for a variety chemicals and has been used in both laboratory studies and clinical practice [218].

In light of the liposome-based designs, Miyabe’s group has reported that the YSK05-based lipid particles were able to entrap and deliver cyclic di-GMP into the RAW 264.7 macrophage cell line [219] (Figure 6). These particles induced cellular expression of type I interferon genes which were effectively blocked by the TBK1 inhibitor BX795, suggesting that the interferon response was specifically induced via the STING/TBK1 pathway. Furthermore, the YSK05-based particles also express high levels of the antigen-presenting molecule MHC class I and T cell co-stimulating molecules CD80 and CD86 that are a prerequisite of T lymphocyte activation; all of these characteristics suggest they offer potential as adjuvants in anti-tumour therapies. Preliminary tests carried out by Miyabe and colleagues showed that mice immunized with cyclic di-GMP containing YSK05-liposomes reject tumour implantation compared to matched controls [219]. Subsequently, Nakamura’s group demonstrated that in vivo injection of these particles greatly enhances the expression of IFN-I and MHC class I molecules in tumourigenic mice, resulting in augmented NK cell activation and potent innate immune protection against lung melanoma metastasis [220]. Therefore, the YSK05 liposomes offer a potential vehicle to assist delivery of STING ligands and to develop STING-based adjuvants for cancer immunotherapy.

As an alternative approach, a nanoparticle-based delivery system developed by Lee and colleagues also enables in vitro delivery of cyclic dinucleotides to target STING pathways for anti-tumour effects [221] (Figure 6). This method employs polyethyleneimine / hyaluronic acid (LH) -based hydrogels to enclose dinucleotide drugs into micron size spheres, which are selectively taken up by phagocytic cells such as RAW 264.7 macrophages, L929 fibroblasts and bone marrow-derived macrophages (BMDMs), but not by non-phagocytic fibroblasts. Thus, the micron-sized particles appear to target phagocytosis specifically to gain entry into the cytoplasm. Furthermore, Lee et al. showed that LH-cGAMP hydrogel can induce IFN-I spikes in RAW264.7 macrophages, which were more than twice the magnitude of that induced by lipofectamine-delivered cGAMP at the same dose [221]. Administration of ovalbumin-containing LH-cGAMP particles in mice activates both the type I interferon response and humoral production of IgG to protect against ovalbumin challenge. Taken together, Miyabe and Lee’s work indicates that the modified liposome-based delivery systems can markedly enhance in vitro and in vivo delivery of cyclic dinucleotide to cytoplasmic STING, and this success will promote the development of novel cancer vaccination and immunotherapies dependent on STING signalling.

## Conclusions

Cytoplasmic DNA has been implicated in many human pathologies, many associated with chronic inflammation. Research has revealed that the ER transmembrane protein STING is a crucial player in dsDNA pathogen -sensing pathways whose dysregulation contributes to the development of several diseases. By responding to DNA sensors and cyclic dinucleotides, STING induces IRF3- and NF-κB –dependent pathways to elicit proinflammatory responses against infection and cancer progression. STING also directly crosstalks with several other regulators to modulate critical biological processes including autophagy and cholesterol biosynthesis. It is evident that activating STING results in the type I interferon response to protect against infection and tumour formation, while dysregulated gain-of-function STING mutations lead to detrimental consequences of autoimmunity. Understanding the molecular signalling mechanisms of STING activation has provided new insights to advance therapeutic strategies in treating infection, cancer and autoimmune diseases. It has also prompted the development of intracellular delivery systems to administer STING agonists. Despite the fact that STING has only been studied for a decade, this adaptor protein will continue to attract attention in immunology research and clinical practice into the future.

## Abbreviations

ADAR1: Adenosine deaminase acting on RNA 1
AGS: Aicardi-goutières syndrome
AMFR: Autocrine motility factor receptor
AMPK: Adenosine monophosphate activated protein kinase
ANA: Antinuclear antibody
Atg: Autophagy-related gene
BMDM: Bone marrow-derived macrophage
CDN: Cyclic dinucleotide
cDNA: Complementary DNA
cGAMP: Cyclic guanosine monophosphate – adenosine monophosphate; cyclic GMP-AMP
cGAS: Cyclic guanosine monophosphate – adenosine monophosphate synthase; cyclic GMP-AMP synthase
DAI: DNA-dependent activator of IFN-regulatory factor
DC: Dendritic cell
DDX41: DEAD-Box helicase 41
DENV: Dengue virus
DNA: Deoxyribonucleic acid
dsDNA: Double-stranded DNA
ER: Endoplasmic reticulum
ERAD: Endoplasmic reticulum-associated degradation
FCL: Familial chilblain lupus
GM-CSF: Granulocyte-macrophage colony-stimulating factor
HAQ mutant: R71**H**-G230**A**-R293**Q** mutant
HCMV: Human cytomegalovirus
HIV: Human immunodeficiency virus
hSTING / mSTING: Human STING / mouse STING
HSV: Herpes simplex virus
IFI61: IFNγ-inducible protein 16
IFIH1: IFN-induced helicase C domain containing protein 1
IKKα/β: IκB kinase α/β subunits
IL-1α: Interleukin-1α
INSIG1: Insulin-induced gene 1
IRF: Interferon regulatory factor
ISG: Interferon-stimulated gene
ISGF3: Interferon-stimulated gene factor 3
JAK: Janus kinase
LH: Polyethyleneimine / Hyaluronic acid
MAMs: Mitochondria-associated membranes
MAVS: Mitochondrial antiviral signalling protein
MDA5: Melanoma differentiation-associated protein 5
MHC class I: Major histocompatibility complex class I
microRNA: miR
MLV: Murine leukemia virus
mtDNA: Mitochondrial DNA
NF-κB: Nuclear factor kappa-light-chain-enhancer of activated B cells
NLR: NOD-like receptor; nucleotide-binding oligomerization domain-like receptor
PDE: Phosphodiesterase
PD-L1: Programmed death ligand 1
Pol III: RNA polymerase III
RLR: RIG-I like receptor; retinoic acid-inducible gene I –like receptor
RNA: Ribonucleic acid
RNase: Ribonuclease
RNF5: RING finger protein 5
SAMHD: SAM domain and HD domain
SAVI: STING-associated vasculopathy with onset in infancy
SIV: Simian immunodeficiency virus
SLE: Systemic lupus erythematous
SNP: Single nucleotide polymorphism
ssRNA: Single-stranded RNA
STAT: Signal transducer and activator of transcription 1
STING: STimulator of INterferon Genes
TBK1: TANK binding kinase 1
TNF-α: Tumour necrotic factor-α
TRAP: Translocon-associated protein
Treg: Regulatory T cells
TREX1: 3′ repair exonuclease 1
TRIM56: Tripartite motif containing 56
Tyk: Tyrosine kinase
Type I interferon/ interferon: IFN-I/ IFN
VSV: Vesicular stomatitis virus
XBP-1: X-Box binding protein 1
